# The application of antibody-based agents in cancer therapy based on their mechanisms of action

**DOI:** 10.1016/j.fmre.2024.02.021

**Published:** 2024-04-15

**Authors:** Kewen Qian, Guangyao Li, Shuyi Zhang, Yitan Zou, Hongru Ai, Xinya Zheng, Wenyan Fu, Changhai Lei, Shi Hu

**Affiliations:** a Department of Biomedical Engineering, College of Basic Medical Sciences, Second Military Medical University, Shanghai 200433, China; b Department of Biophysics, College of Basic Medical Sciences, Second Military Medical University, Shanghai 200433, China; c Department of Respiratory and Critical Care Medicine, the First Affiliated Hospital of Second Military Medical University, Shanghai 200433, China; d Department of Assisted Reproduction, Shanghai Ninth People's Hospital, Shanghai Jiao Tong University School of Medicine, Shanghai 200011, China

**Keywords:** Antibody therapy, Cancer therapy, Antibody-drug conjugates, Immunotherapy, Monoclonal antibodies

## Abstract

Monoclonal antibodies and antibody-based agents are undergoing remarkable development in cancer immunotherapy. Based on their structural components, these antibodies mediate the crosslinking of tumor cells and the immune system, enabling specific antigen targeting and broad immune responses. Novel engineering strategies are aimed at improving both specificity and affinity of antigen recognition and enhancing the recruitment and activation of immune cells. Antibody-based therapeutics have been expanded for use in combination with other immunotherapies such as cellular immunotherapy and therapeutic vaccines. In this review, we provide comprehensive updates on the ongoing clinical development and evolving practices in the field, with a specific emphasis on the intricate mechanisms of action.

## 1 Introduction

The last three decades have marked noteworthy milestones in the evolutionary trajectory of antibody therapy, signifying substantial advancements. Trastuzumab, the pioneering humanized monoclonal antibody (mAb) targeting the tumor biomarker HER2, represents a breakthrough therapeutic modality of solid malignancies in 1998 [1]. In 2000, the advent of the first ADC realized the integration of specific antibody targeting with toxic chemical agents, offering a novel therapeutic approach with great potential [2]. Furthermore, catumaxomab, the first bispecific antibody approved in 2009, played an instrumental role in enhancing the effectiveness of antibodies and addressing drug resistance [3]. Since the approval of CTLA-4 mAb in 2011, the subsequent introduction of several PD-1 mAbs in the market has significantly reinforced the pivotal role of immune checkpoint inhibitors (ICIs) in the realm of clinical cancer therapy. Presently, antibody-based drugs account for nearly one-fifth of new drug approvals by FDA each year, demonstrating their escalating market share and significance [4].

**Fig. 1:**
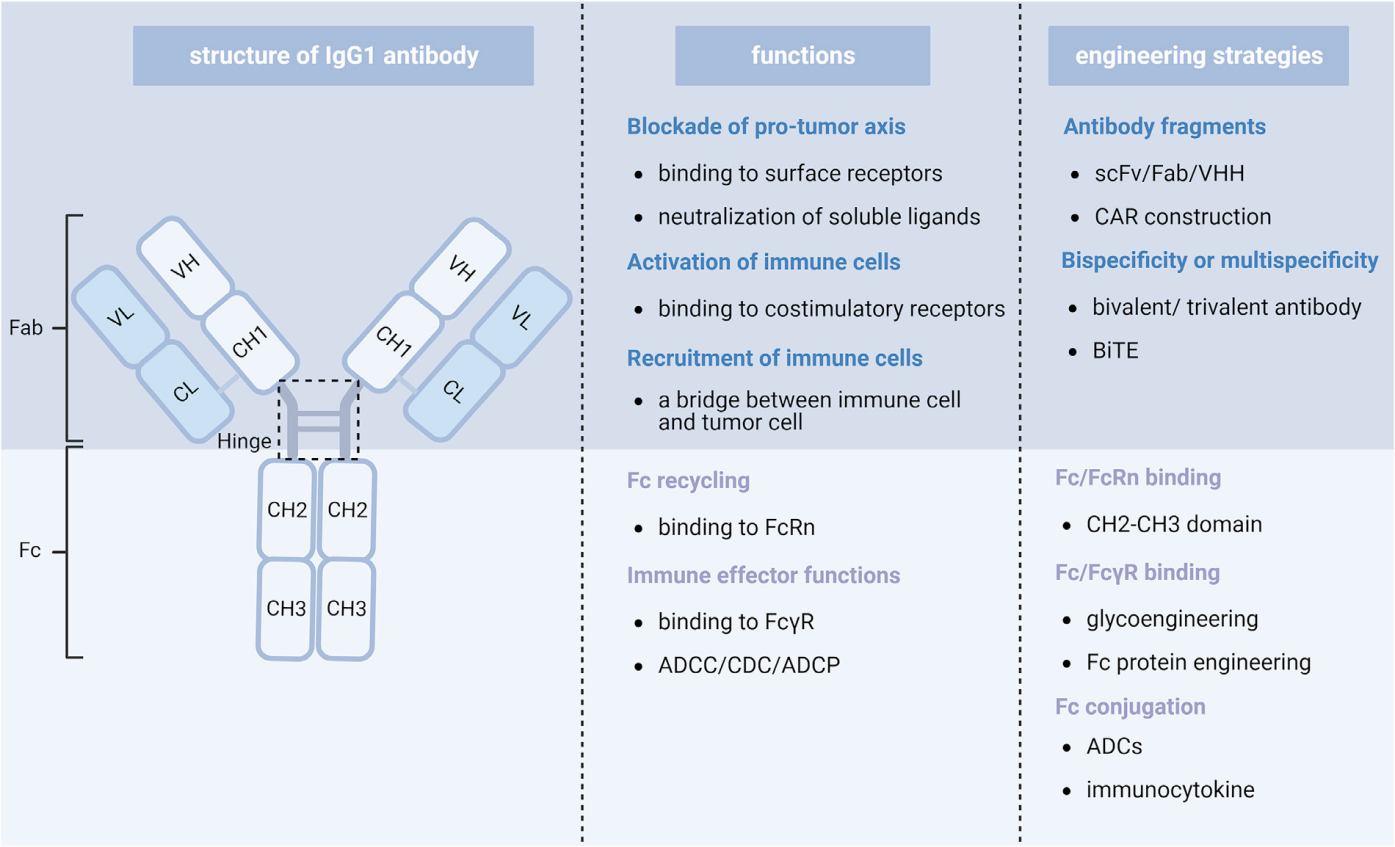
**An overview of the molecular structure, antitumor mechanisms, and engineering strategies of IgG antibodies**.

Through advancements in antibody engineering techniques and exploration of novel targets, the biological properties and therapeutic actions of therapeutic antibodies have experienced notable progress [5,6]. The development of antibody preparation methodologies, such as phage display, has successfully transformed therapeutic antibodies from murine-derived to fully humanized, effectively mitigating their immunogenicity [5]. Additionally, antibody fragments and the design of alternative bioactive forms, including single-chain fragment variable (scFv), antigen-binding fragment (Fab), minibodies, nanobodies, antibody-drug conjugates (ADCs), bispecific and multispecific antibodies, T-cell receptor-like antibodies (TCRm), and chimeric antigen receptors (CARs), has considerably broadened the therapeutic potential of antibody-based agents [7] (Fig. 1). Particularly, the paradigm surrounding the therapeutic mechanisms mediated by these antibodies has shifted beyond mere independent activation or blocking effects. Increasing attention has been devoted to extensively exploring immune responses, such as the activation of diverse immune cells and the regulation of the immune microenvironment, as demonstrated by a growing body of both preclinical experimentation and clinical data. These findings align with the development trajectory of cancer immunotherapy and signify a broader perspective on the therapeutic effects of these antibody-based interventions [8,9].

Among the five distinct isotypes of human antibodies, the immunoglobulin G (IgG) isotypes and their corresponding antibody fragments, notably IgG1 and IgG4, hold prominent utility for therapeutic purposes [10]. The human IgG molecule, comprising two heavy and two light polypeptide chains, which are connected by disulfide bonds, forms a protein complex with an approximate molecular weight of 150 kDa. The heavy chain encompasses a variable domain (VH) and constant domains, namely CH1, CH2, and CH3 fragments (Fig. 1). Notably, the binding of the CH2 and CH3 fragments, known as the Fc domain, with the neonatal Fc receptor (FcRn) expressed on the surface of vascular endothelium governs essential circulatory properties, including relatively slow tissue penetration and extended serum half-lives [11]. Additionally, their interaction with Fc receptors (FcRs) present on diverse innate immune effector cells triggers a broad array of Fc-mediated immune effector functions, such as antibody-dependent cellular cytotoxicity (ADCC), antibody-dependent cellular phagocytosis (ADCP), and complement-dependent cytotoxicity (CDC) [8]. The light chain of the antibody consists of a variable domain (VL) and a single constant domain, which forms a disulfide linkage with the CH1 domain of the heavy chain. As the two variable domains recognize and bind to the antigen's epitope, they determine the affinity and specificity of the monoclonal antibodies (mAbs) toward their targeted tumor antigens [10].

The therapeutic mechanisms of antibody-based agents in cancer therapy can be primarily classified into two distinct groups, delineated by their structural components (Fig. 1). The first category encompasses immune effector-independent mechanisms, wherein full-length IgG mAbs, antibody fragments without a Fc domain, as well as antibodies that are conjugated with a cytotoxic drug exhibit direct cell-targeted activation, apoptosis or neutralization effects. These approaches aim to disrupt ligand-receptor interactions or exploit the target-specific properties of antibodies. The second category comprises immune effector-required mechanisms, wherein full-length IgG mAbs, bispecific T cell engagers (BiTE), chimeric antigen receptor (CAR)-based immune cells, as well as ADCs, aim to engage extensive composition of the immune system and broadly modify the whole tumor microenvironment (TME).

## 2 Direct cytotoxicity on targeted cells and stimulation of effector immune cells

### 2.1 Induction of cell apoptosis

**Fig. 2:**
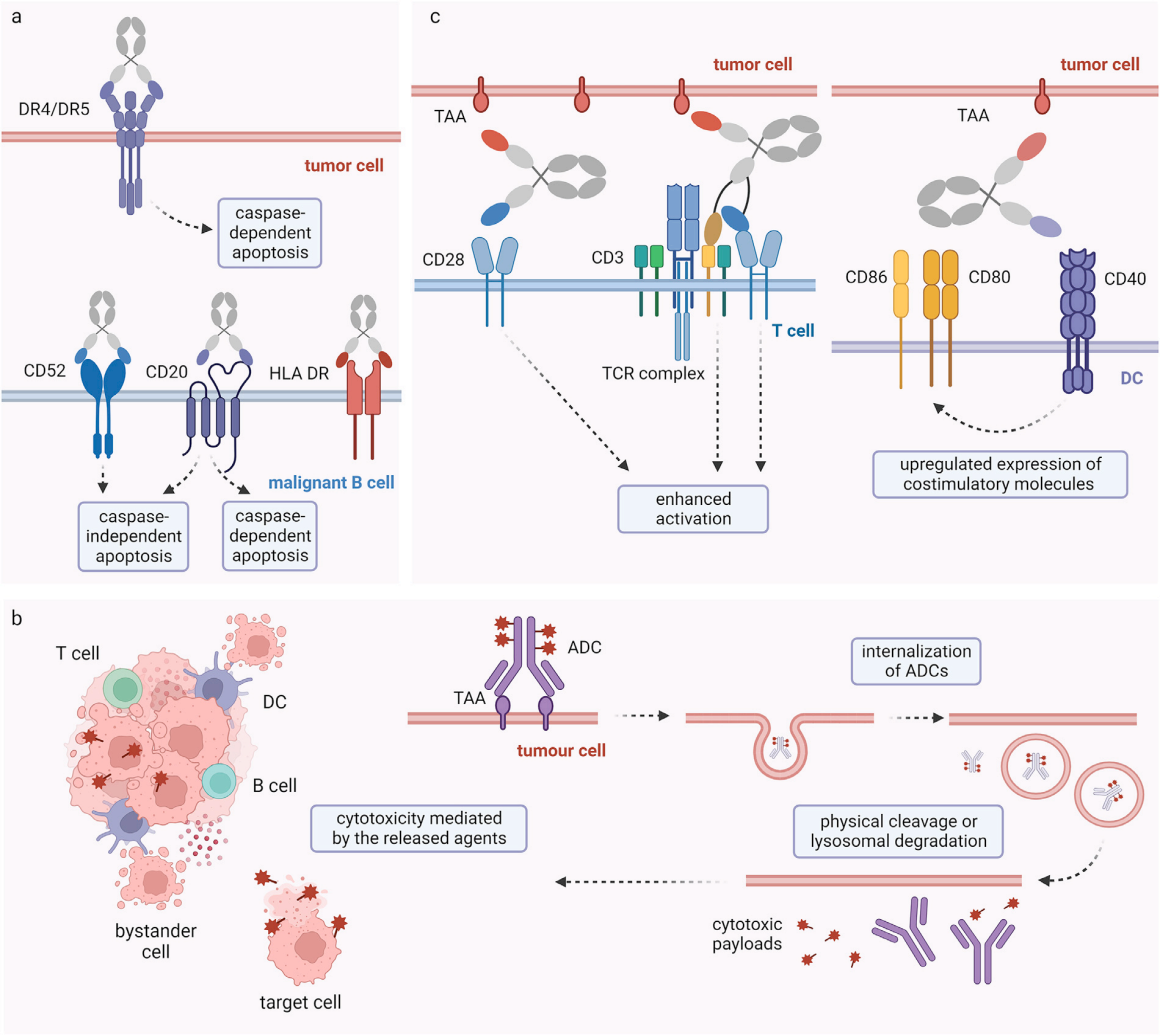
**Antibodies act as direct antagonists to tumor cells or agonists to immune cells.** (a) Induction of tumor cell apoptosis based on caspase-dependent or caspase-independent mechanisms. (b) The internalization of ADCs and release of toxic payloads after binding to targeted antigens and further cytotoxicity to bystander tumor cells via extensive activation of immune responses. (c) Engagement and activation of T cells through bispecific and multispecific CD28 agonistic antibodies and promotion of DC mature through bispecific CD40 agonistic antibodies.

Without the help of immune elements like immune cells and cytokines, the cytotoxic effect of mAbs on tumor cells is primarily achieved through the induction of intracellular apoptosis. This process can be further categorized into two distinct subtypes based on the triggering mechanism: caspase-dependent and caspase-independent apoptosis (Fig. 2a). Upon the antibody binds to the corresponding receptors as an agonist, it can trigger a series of apoptotic signaling pathways through the intracellular domains, resulting in spontaneous apoptosis of target cells.

In a clinical trial utilizing rituximab for B-CLL treatment, analysis of leukemia cells derived from patients revealed the activation of caspase-9, caspase-3, and poly (ADP-ribose) polymerase (PARP) cleavage [12]. The interaction between death receptors expressing on the surface of tumor cells and their ligands transmits caspase-dependent apoptosis signals through the intracellular death domain. The interaction between tumor necrosis factor-related apoptosis-inducing ligand (TRAIL) and death receptors DR4 (TRAIL-R1) and DR5 (TRAIL-R2) can transmit caspase-dependent apoptotic signals via their intracellular death domain. Antibodies have been designed to artificially activate DR4 and/or DR5. Among these, mapatumumab, an agonistic DR4 antibody, has progressed to phase II clinical trials. However, its clinical efficacy has thus far demonstrated significant success solely in follicular non-Hodgkin's lymphoma cases [13]. A potential concern contributing to disappointing outcomes could be the insufficient ability of these agents to induce TRAIL receptor clustering, which is a crucial step for the induction of apoptosis with optimal efficacy. To address this problem, various derivatives, including scFvs, bispecific antibodies, and CARs have been designed to enhance the activation of TRAIL receptors [14].

Caspase-independent apoptosis, which relies on actin-dependent mechanisms, is frequently observed in various studies. For instance, the parental antibody of obinutuzumab, an anti-CD20 ADC, has been demonstrated to induce programmed cell death (PCD) in malignant B cells through a caspase-independent pathway [15]. Further investigations involving obinutuzumab and apolizumab, an anti-HLA DR mAb, have explore their ability to elicit PCD. The induction of PCD in these cases was found to be dependent on the generation of reactive oxygen species (ROS) derived from nicotinamide adenine dinucleotide phosphate (NADPH) oxidase, while caspase activity played a negligible role [16,17]. Caspase-independent cell death induced by alemtuzumab, a humanized IgG1 mAb against CD52, was mediated by the formation of large CD52 and ganglioside GM-1-enriched domains within the plasma membrane, necessitating F-actin cytoskeletal rearrangement, and inhibited by depletion of membrane cholesterol [18]. Anti-CD47 mAbs were found to function in a caspase-independent manner in B-CLL, as the addition of caspase inhibitors failed to prevent the cytoplasmic events of apoptosis [19].

### 2.2 Cytotoxicity mediated by cytotoxic payloads of ADCs

ADCs have emerged as a prominent area of focus within the realm of cancer immunotherapy. There are currently 16 ADCs approved by the Food and Drug Administration (FDA) worldwide, and this number has not stopped updating in recent years. ADCs are immunoconjugates wherein a monoclonal antibody is linked to a cytotoxic drug through a chemical linker. Following the binding of the ADC to the specific target antigen, internalization of the ADC is facilitated through the process of endocytosis, thereby enabling the subsequent liberation of the cytotoxic payload through either physical cleavage or lysosomal degradation. The liberated payloads exert their toxic effects, leading to tumor cell death [20,21] (Fig. 2b).

The primary advantages of ADCs lies in their ability to selectively recognize tumor antigens, thereby enhancing the efficacy and safety of the cytotoxic payloads, while individual administration of these payloads and their systemic exposure often result in excessive toxicity [22]. According to their mechanisms of action, these payloads can be classified into two categories: one is the tubulin inhibitors, which impede tubulin polymerization, resulting in cell cycle arrest at the G2/M phase and subsequent initiation of cell apoptosis, the other is the DNA-damaging agents, which bind to DNA's minor groove and induce cell death through DNA cleavage, DNA alkylation, or disruption of DNA replication [23,24]. Currently widely utilized cytotoxic agents in ADCs include monomethyl auristatin E and F (MMAE and MMAF) [25], [26], [27], calicheamicin, and pyrrolobenzodiazepines (PBDs) [28]. The relevant information has also been well reviewed elsewhere [29].

As we focus on the antibody component of ADCs, two crucial factors warrant careful consideration. Firstly, the targeted antigen must possess high specificity toward tumor tissues, thereby minimizing the occurrence of “on-target, off-tumor” toxicity. Secondly, the targeted antigen should facilitate internalization via endocytosis, enabling the subsequent release of toxic payloads [30]. Within the realm of hematological malignancies, lineage-specific markers like CD22, CD33, and CD30 have emerged as popular choices for target antigens. For instance, brentuximab vedotin, an ADC comprising an anti-CD30 mAb linked to MMAE through a protease-cleavable linker, has obtained FDA approval for treating patients with relapsed or refractory (R/R) CD30-positive Hodgkin lymphoma. It is currently being explored in various treatment combinations for hematological malignancies [30,31]. In the field of solid tumors, attention is directed to overexpressed antigens that are well demonstrated. Take HER2 for example, trastuzumab emtansine, featuring the microtubule inhibitor DM1, is the first anti-HER2 ADC approved for the treatment of solid tumors in 2013 [32]. A novel generation anti-HER2 ADC, trastuzumab deruxtecan, has achieved significant success, which may represent a breakthrough in both HER2-positive solid tumor therapy and ADC development. In 2019, it was initially approved by FDA for HER2-positive breast cancer based on the promising clinical outcomes (NCT03248492) in patients resistant to previous anti-HER2 therapy [33]. More recently, FDA granted accelerated approval to trastuzumab deruxtecan for unresectable or metastatic non-small cell lung cancer (NSCLC) after the publication of its clinical data (NCT03505710) in 2022 [34]. Trastuzumab deruxtecan exhibited a significant improvement in overall survival compared to trastuzumab emtansine (NCT03529110) [35]. Notably, clinical trials exploring the association between HER2 expressing levels and therapeutic efficacy suggest unknown mechanisms of trastuzumab deruxtecan. A phase III trial (NCT03734029) involving metastatic breast cancer patients with low HER2 expression exhibited significantly longer progression-free and overall survival than chemotherapy [36]. In a phase II DAISY trial (NCT04132960), the objective response rate in patients with HER2-low and HER2 non-expressing metastatic breast cancer was 37.5% (95% confidence interval (CI) 26.4–49.7) and 29.7% (95% CI 15.9–47), respectively, compared to 70.6% in HER2 overexpressing patients (95% CI 58.3–81), suggesting additional mechanisms rather than the determinant HER2 expression [37].

In addition to their cytotoxic potential, the initial destruction of targeted tumor cells can lead to subsequent damage to bystander tumor cells and stromal tissue as a result of more extensive immune responses (Fig. 2b). The provocation of extensive cellular death, either by apoptosis or alterative mechanisms, has demonstrated the liberation of a wide array of antigens that elicit immunogenic cell death, subsequently stimulating broad immune responses [38,39], including the activation of dendritic cells (DCs) and enhanced recruitment of CD8+ effector T cells [40,41]. The activation of the proinflammatory TME through ADCs presents an opportunity for their application in combination with other immunotherapies, especially immune checkpoint inhibitors (ICIs). A number of clinical trials have validated the efficacy and safety of anti-PD-1 antibodies and ADC administration [42], [43], [44]. Actually, as inadequate T cell infiltration due to lack of tumor immunogenicity plays a crucial role in the resistance to PD-1/PD-L1 blockade, this may be one of the mechanisms by which ADCs contributes to ICI treatment. An alternative approach is to construct ADCs composed of anti-PD-1/PD-L1 antibody. For instance, when an anti-PD-L1 antibody is conjugated to doxorubicin using a hydrazone linker, the disruption of the tumor extracellular matrix can facilitate the penetration of these anti-PD-L1 ADCs into the tumor site [45].

Additionally, it is noteworthy that the antibody component of ADCs primarily originates from an IgG1 mAb, and the antitumor effect of ADCs is, in part, reliant on Fc-mediated immune effects such as ADCC, ADCP, and CDC [46,47]. The Fc region of ADCs can be engineered to modulate these Fc-mediated effects. For instance, MEDI4276, an ADC composed of a tetravalent mAb that binds to two distinct HER2 epitopes, involved a lysine-to-phenylalanine substitution to eliminate FcγR interactions with the aim of avoiding adverse reactions in circulation [48].

In fact, the direct toxicity of tumor cells mediated by the above two therapeutic antibodies is more characteristic of traditional chemical intervention, of which the recruitment of other immune cells are not required for therapeutic efficacy. However, the property of antibodies confer them targeting specificity that is absent in other therapies. Particularly, ideal ADCs can carry cytotoxic agents with higher toxic potency compared to conventional chemotherapeutics [20]. In addition, localized tumor cell apoptosis can activate more extensive immune components, which may attribute to more than direct killing and such mechanisms of action deserve further investigation for combined immunotherapy regimen. Being a role as immune mediators, antibodies can indirectly induce a wider range of immune responses, such as activating the cytotoxicity of adaptive immune cells. Such mechanisms of action tend to be more complex and have great potential for engineering design.

### 2.3 Activation of immune cells by agonistic antibodies

IgG antibodies can act as agonists to deliver enhanced costimulatory signals to active immune cells (Fig. 2c). Monoclonal antibodies have been developed to direct towards the principle costimulatory domains CD28 and CD40. CD28 is a costimulatory receptor commonly expressed on T cell surface. After binding of CD28 agonists, activated intracellular signaling of TCRs can induce the release of cytotoxic molecules and cytokines. The activation of CD40 agonists can upregulate the expression of costimulatory receptor CD80/86, which is closely related to the maturation of dendritic cells (DCs), resulting in enhanced antigen presentation and recruitment of adaptive immune cells. The research progress on CD28 has faced setbacks due to severe adverse reactions observed in an early clinical trial of an anti-CD28 mAb, TGN1412, leading to the interruption of subsequent clinical studies with a reduction in drug dosage. Within 90 min after receiving a single intravenous dose of the drug, all six participants had a systemic inflammatory response syndrome, and unexpected depletion of lymphocytes and monocytes was observed within 24 h after infusion [49]. Subsequent analyses proposed that excessive T cell activation and cytokine release syndrome (CRS) are associated with excessive doses calculated from in vivo experiments, the immune environment and drug responses of human are very different from those of animals [50]. Fortunately, CD28 has been reevaluated as a potential target in light of the discovery of novel immune regulatory mechanisms. In 2017, a study revealed that, rather than solely inhibiting TCR activation, the CD28/B7 costimulatory pathway is crucial for the effectiveness of anti-PD-1 therapy in chronic viral infections [51]. The mechanism has been further supported by the development of bispecific antibodies that cross-link a tumor-specific antigen (TSA) with CD28 (TSA × CD28). These bispecific antibodies have exhibited synergistic effects when combined with anti-PD-1 antibodies, leading to the establishment of long-term immune memory against tumors in genetically humanized immunocompetent mouse models [52,53]. More recent findings highlighted the potential of combining a CD22- and CD28-bispecific antibody, REGN5837, with odronextamab, a CD20- and CD3-bispecific antibody, to enhance the antitutmour activity of odronextamab [54]. Trispecific antibodies aim to leverage the costimulatory effects of both CD3 and CD28. A trispecific antibody designed to engage CD38, CD3 and CD28 has been shown to facilitate efficient T cell stimulation. The anti-CD38 domain directs T cells toward myeloma cells, certain lymphomas, and leukemias [55]. Another trispecific antibody, exhibiting specificity for HER2, CD3 and CD28, was demonstrated to elicit regression of breast cancers in a humanized mouse model and has been employed in clinical investigations [56]. Notably, as these bispecific and trispecific antibodies refer to the design of CD3-targeting bispecific T cell engagers (BiTEs), the recruitment of T cells occupies another crucial immune effect in addition to CD28-mediated T cell activation.

Different from CD28 agonistic antibodies, CD40 agonistic antibodies raise an important concern regarding secondary crosslinking through FcγR. CD40/CD40L interaction can trigger DC activation and enhance antigen presentation via upregulated expression of CD80/86 that further interact with T cells. Multiple studies have provided evidence highlighting the essential role of Fc-mediated effects for anti-CD40 antibodies. In a study, an anti-CD40 mAb with enhanced FcγRIIb binding exhibited greater antitumor responses [57]. The activation of nuclear factor-κB (NFκB) activity in B cells relies on Fc/FcγR interactions of CD40 agonistic antibodies [58], and the stimulatory effect of FcγRIIb was mediated through antibody cross-linking delivered in trans between neighboring cells [59]. Despite the evident effects of CD40 agonists, their clinical benefit remains relatively minimal [60,61], as well as CD40 agonist antibodies. ABBV-428 is a bispecific antibody targeting both mesothelin and CD40, and its phase I clinical data exhibited limited clinical efficacy among patients with advanced mesothelioma or ovarian cancer [62]. However, the combination of CD40 agonist antibodies and other immunotherapies, such as CAR-T cell therapy and ICI therapy, has revealed more favorable outcomes [61]. Studies have highlighted the therapeutic potential of combination of CD40 agonists and anti-PD-1 mAbs in reshaping immune-resistant tumors [63,64].

## 3 Blockade of receptors or neutralization of ligands: interference of pro-tumor signals

Complex pathways are involved to support the infinite proliferation and late metastasis of malignant tumor cells. Especially in the field of solid tumors, the tumor microenvironment (TME) consists of tumor cells, immune cells, stromal components, and various cytokines. Multiple tumor-promoting factors permeate the TME, which can be categorized into several mechanisms [65], [66], [67]. First, activation of the receptors on tumor cell surface can directly stimulate intracellular growth and inflammatory signaling pathways. Second, activation of signals providing nutritional support such as the vascular endothelial growth factor (VEGF)/ VEGF receptor (VEGFR) pathway creates a favorable condition for tumor cell proliferation. Third, released immunosuppressive cytokines can bind to corresponding receptors on the surface of immune cells, activate suppressive pathways and impair their immune activity. In addition, under the pressure of targeted therapy, immune escape of the tumors, which is manifested by downregulated expression of targeted antigen and upregulation of immune checkpoint signals can seriously damage the function of immune cells. Therefore, neutralizing the ligands or blocking the receptors of relevant signaling axis is an efficient approach to inhibit tumor growth and restore the activity of immune cells.

### 3.1 Blockade of growth signals that promote tumor growth and metastasis

As for blocking of membrane receptors, we take the ErbB family as an example. The epidermal growth factor receptor (EGFR) and human epidermal growth factor receptor 2 (HER2) serve as frequently utilized targets of antibodies and have shown promising antitumor effects [68]. In addition to homodimers, HER2 can form heterodimers with other receptors of the HER family and overamplify their signals. After dimerization and phosphorylation of the intracellular domains, further downstream signaling cascades are activated, such as the Ras/Raf/mitogen-activated protein kinase (MAPK), the phosphoinositide 3-kinase/Akt, and the phospholipase Cγ (PLCγ)/protein kinase C (PKC) pathways that promote cell growth and survival and cell cycle progression [69]. Trastuzumab has been early utilized in the management of early-stage HER2-positive breast cancer, through its binding with the extracellular domain (ECD) of HER2. Trastuzumab obstructs both homodimerization and heterodimerization, consequently suppressing intracellular HER2 signals and inhibiting cell cycle progression [70]. Pertuzumab is another humanized anti-HER2 monoclonal antibody, which targets a distinct ECD domain of HER2, effectively preventing HER2 heterodimerization [71]. The combination of trastuzumab and pertuzumab has also been investigated in various treatment regimens, exhibiting promising results. One of the advantages is that in addition to blocking HER2 signals, their synergy can also enhance Fc-mediated immune effects, which we will mention below [72]. From the classical anti-EGFR antibodies, such as Cetuximab, there are some more interesting mechanisms in addition to signal blocking, as the internalization of the antibody-receptor complex can overall downregulates EGFR expression level, leading to a sustaining antitumor effect [73]. Cetuximab can also induces cell apoptosis and decrease the production of vascular endothelial growth factor (VEGF) [74].

**Fig. 3:**
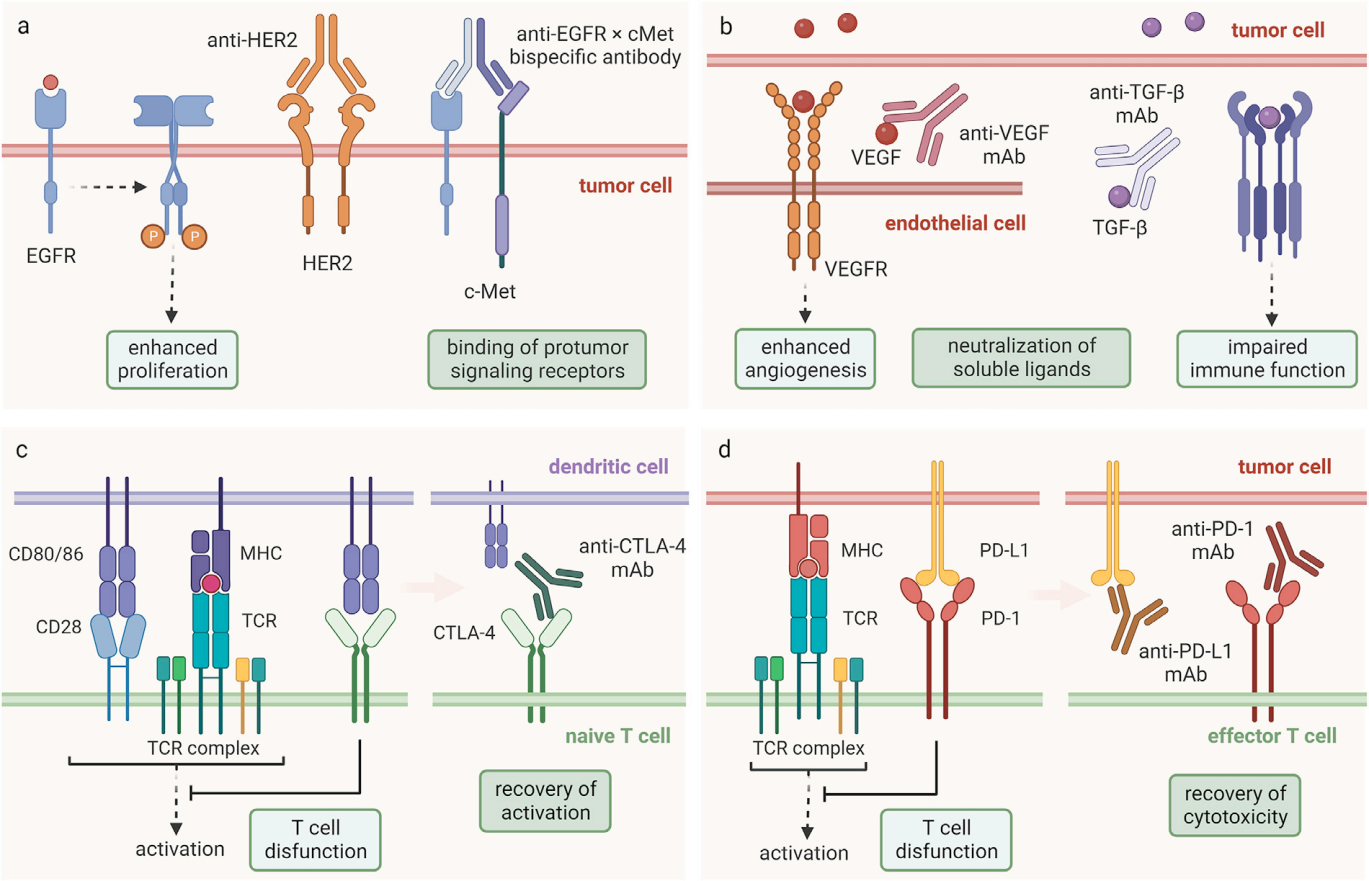
**Blockade of protumor ligand/receptor interactions.** (a) Competitively binding of the surface receptors and blockade of signaling pathways that promote tumor growth via monoclonal antibodies or bispecific antibodies. (b) Neutralization of soluble protumor signaling ligands and immunosuppressive cytokines. (c, d) Mechanisms of T cell immunosuppression mediated by CTLA-4 and PD-1/PD-L1 signaling and the diagram of immune checkpoint blockade to revise T cell activation.

Neutralization of soluble ligands is another consideration. After binding of their receptors, VEGF, cytokines, and chemokines can contribute to the facilitation of angiogenesis, extracellular matrix fibrosis, and the migration of tumor cells through dynamic crosslinking (Fig. 3b). In a hypoxic environment, tumor cells can promote tumor angiogenesis by releasing VEGF to create an immature and disorganized vascular network and get more blood supply [75]. Monoclonal antibodies against VEGF aim to sequester circulating VEGF and prevent its binding to the VEGF receptor (VEGFR), thus preventing formation of new blood vessels [76]. Such mature antibodies with FDA approval include Bevacizumab, a monoclonal antibody that targets VEGF-A and Aflibercept, a recombinant protein composed of the binding domain of VEGFR fused with the Fc region of human IgG1. Transforming growth factor-β (TGF-β) signaling has gained considerable attention as a pivotal avenue for enhancing the effectiveness of immunotherapies. Released by cancer cells, stromal fibroblasts, as well as other TME constituents, TGF-β can induce uncontrolled proliferation of epithelial cells, drive a deregulated wound-healing program in cancer-associated fibroblasts [77] and suppress the antitumor functions of both innate and adaptive immune cells [78]. Fresolimumab, a humanized IgG4 monoclonal antibody exhibiting neutralization of all three isoforms of TGF-β, has exhibited promising outcomes in a phase I clinical trial [79]. Next-generation TGF-β-blocking antibodies are tailored to specific TGF-β isoforms. A phase I evaluation of an antibody selectively blocking activated TGF-β1, while sparing TGF-β2 and TGF-β3, has been completed [80], and NIS793, an anti-TGF-β1/β2 antibody, is currently undergoing assessment in patients with advanced cancers [78].

It is worth noting that specific blockade of a pro-tumor signaling cascade can lead to upregulation of alterative dominant antigens or pathways, as well as immunosuppressive signals, which is driven by intratumor heterogeneity (ITH) and acquired resistance under therapeutic pressure [81,82]. For instance, the VEGF and EGFR pathways exhibit overlapping downstream signaling cascades and possess the capability to independently drive oncogenesis [83]. The similarity in downstream pathways between EGFR and HER2 can lead to resistance against anti-EGFR treatments via the upregulation of HER2 expression [84]. Dual targeting strategies have been widely adopted to mitigate antigen escape. Amivantamab is an Fc-enhanced EGFR and cMet bispecific monoclonal antibody, they can eliminate tumor cells through Fc-mediated effects in the presence of either overexpressed EGFR or cMet [85,86]. Coadministration of different monoclonal antibodies can also exhibit synergistical effects by simultaneously blocking different signals. A typical example is the application of relatlimab targeting the lymphocyte activation gene 3 (LAG-3), of which the expression is tied to T cell exhaustion, in combination with nivolumab, a classical anti-PD-1 monoclonal antibody. The combined option has been approved for clinical treatment of melanoma. In comparison to conventional IgG antibodies, monovalent antibody fragments such as scFv and Fab prioritize their antagonistic effects and have the potential to be developed as multivalent antibodies. For instance, a bispecific nanobody against both VEGF and angiopoietin 2 (Ang2) exhibited promising results in a phase IV clinical trial for the treatment of advanced NSCLC when administrated in combination with an anti-PD-1 antibody [87]. However, despite the large number of experimental studies on various antibody fragments, their setbacks such as the short half-life significantly limit their individual application and engineering approaches are required for enhanced performance.

### 3.2 Immune checkpoint blockade

The success of immune checkpoint inhibitors (ICIs) has marked a significant revolution in the field of cancer immunotherapy. Immune checkpoints play a pivotal role as significant immunomodulators, responsible for maintaining peripheral tolerance and regulating the extent and duration of immune responses. However, they can be “hijacked” by cancer cells, resulting in the inhibition of T cell activation and impaired antitumor responses, which can further facilitate tumor escape [88]. Immune checkpoint blockade (ICB) therapy aims to counteract this phenomenon by eliminating the inhibitory signals that suppress T cell activation, thereby reactivating cytotoxic T cells and promoting robust antitumor immune responses, along with the establishment of an inflammatory microenvironment conducive to antitumor effects [88]. Monoclonal antibodies against the checkpoint molecules including cytotoxic T lymphocyte antigen 4 (CTLA4), programmed cell death 1 (PD-1) and PD1 ligand 1 (PD-L1) have achieved ideal clinical outcomes, leading to multiple clinical applications for several cancer types [89] (Fig. 3c, d).

The engagement of CTLA-4 with CD80/86 molecules delivers an inhibitory signal by limiting the interaction of CD80/86 with costimulatory CD28 domain [90]. CTLA-4 expression is swiftly induced upon T cell activation and serves as a regulator of T cell amplitude during the initial stages of immune activation [91]. Ipilimumab is a fully humanized mAb against CTLA-4 and received FDA approval in 2011 for the clinical management of patients with malignant melanoma [92]. The binding of tumor-expressed PD-L1 to T cell-expressed PD-1 hampers T cell proliferation and induces their exhaustion within peripheral tissues and tumor sites [93]. Anti-PD-1 and anti-PD-L1 antibodies gained their first FDA approval in 2016 and have witnessed widespread utilization rapidly [10]. The antitumor effects via blockde of other immune checkpoints are also undergoing clinical investigation; however, mostly of the studies are in their early phase I stage, typically combined with PD-1 or PD-L1 blockade antibodies [94]. Among these novel ICIs, monoclonal antibodies targeting T cell immunoglobulin and ITIM domain (TIGIT) and lymphocyte-activation gene 3 (LAG3) have achieved the most advanced clinical progress and are currently undergoing phase III trials [95,96].

In addition to targeting immunosuppressive signals, Fc-mediated immune effects has gained consideration in their action of mechanisms. Anti-PD-1 mAbs are commonly engineered to exhibit impaired binding to Fcγ receptors (FcγRs), thereby focusing on the blockade of PD-1 binding to its ligands, while Fc-mediated effects have been found to contribute to the antitumor activity of anti-CTLA-4 mAbs. Notably, ipilimumab carrying FcγRIIIa-158 V mutants demonstrated a higher response rate compared to individuals with FcγRIIIa-158F homozygotes, suggesting the impact of Fc-mediated effects in enhancing therapeutic outcomes [97]. These Fc-mediated effects have been associated with the depletion of intratumoral regulatory T cells (Tregs) by an anti-CTLA-4 antibodies [98,99]. The role of Fc-mediated effects in PD-L1 mAbs remains controversial. In vivo studies have shown a correlation between the engagement of activating FcγRs and the antitumor activity of anti-PD-L1 mAbs within the TME [100], while a JAVELIN study involving patients with renal cell carcinoma suggested that FcγRIIa and FcγRIIIa polymorphisms do not significantly impact the activity of avelumab [101].

Challenges persist in the field of ICIs, which include non-responsiveness in certain cancer types such as glioblastoma and pancreatic cancer, and the emergence of resistance in patients following an initially promising response to treatment. Actually, the condition of patients’ immune system represents influential factors shaping the antitumor responses to ICIs [102]. Sufficient T cell infiltration and a potential immune environment are required for their function, while the immune responses are often impaired in the immunosuppressive tumor microenvironment (TME). Combination therapy strategies have gained considerable attention as a means to disrupt inhibitory factors and induce responsive immune responses. Intratumoral agents like Toll-like receptor (TLR) agonists have been used to modulate intrinsic molecules and pathways associated with tumor heterogeneity, and cytokines and chemotherapeutics have been employed to augment proinflammatory infiltration, thereby enhancing the activity of ICIs [103]. Preclinical investigations have demonstrated the superiority of combination therapy over monotherapy, particularly the involvement of anti-angiogenesis agents in ICI therapy [104]. For instance, axitinib, a VEGF receptor tyrosine kinase inhibitor, has been combined with pembrolizumab and avelumab for the treatment of advanced renal cell carcinoma. Importantly, both of the combinations exhibited more promising effectiveness than sunitinib monotherapy [105,106]. Several clinical trials have indicated that anti-BRAF/MEK therapies exert effects on the TME that synergize with PD-1/PD-L1 inhibitors to enhance antitumor responses [107], [108], [109]. Significantly, the results of a phase III clinical trial have shed light on the efficacy and safety of triple combination therapy comprising atezolizumab (a PD-L1 inhibitor), vemurafenib (a BRAF^V600^ inhibitor), and cobimetinib (a MEK inhibitor) showed favorable safety, tolerance, as well as notably increased progression-free survival in patients with advanced melanoma harboring BRAF^V600^ mutation [109]. In the realm of adaptive T cell therapy, such as CAR-T cell therapy, the involvement of multiple inhibitory signals within the TME can result in T cell exhaustion, with the PD-1 signal being the most extensively studied. Actually, CAR T-cell administration provides an infiltrate capable of triggering immune responses in immunogenically silent tumors, thereby addressing a key issue associated with ICIs, the lack of antitumor immune responses [110]. For instance, in a phase I clinical trial (NCT02414269) using anti-mesothelin CAR T cells, the administration of pembrolizumab consecutively resulted in a median overall survival of 23.9 months with a 1-year overall survival rate of 83%. These promising findings support the initiation of a subsequent phase II clinical trial [111].

## 4 Engagement of immune cells and activation of extensive immune responses

In addition to direct signaling activation or inhibition, cellular immune responses have received increasing attention during the development of antibodies. Rich binding sites of a monoclonal antibody allows it to recruit a variety of immune cells via different mechanisms, and various antibody domains allow for engineering construction and act as a linker between tumor cells and immune cells. Antibodies can activate both innate and adaptive immune responses involving multiple immune cells.

### 4.1 Fc-mediated immune effects

**Fig. 4:**
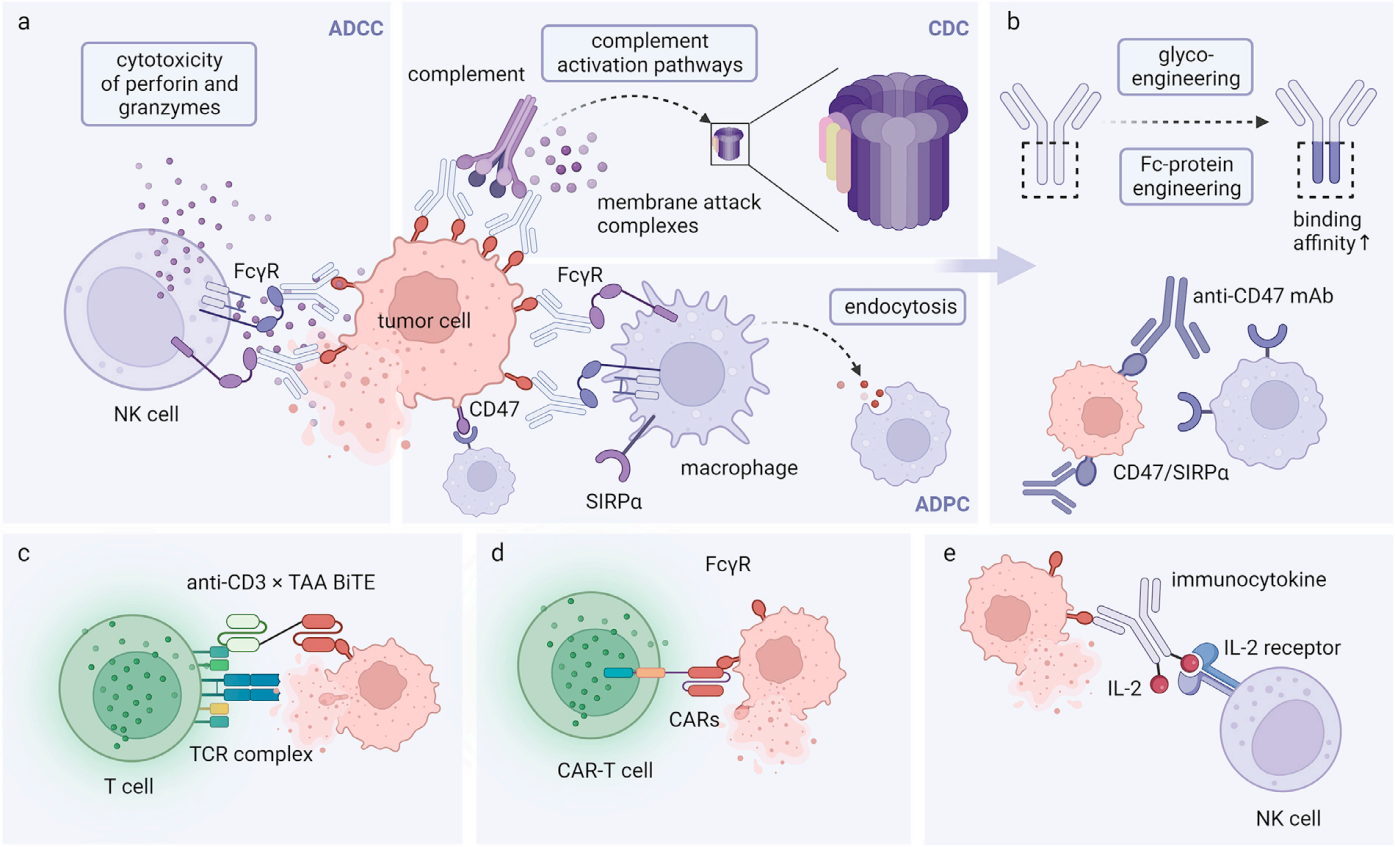
**Immune effector functions mediated by antibody-based agents.** (a) Antibody-dependent cellular cytotoxicity (ADCC), antibody-dependent target cell phagocytosis (ADCP), and complement-dependent cytotoxicity (CDC) mediated by the Fc region of a full-length IgG antibody. (b) Modulation of Fc/FcRn or Fc/FcγR binding affinity through Fc glyco-engineering or Fc protein-engineering approaches. (c) A “bridge” model of bispecific T cell engagers (BiTE). (d) CAR-T cells recognize specific tumor antigens and exert antitumor cytotoxicity.

Antibodies can regulate both innate and adaptive immune responses through the interaction of their crystallizable fragment (Fc) domain and Fcγ receptors (FcγRs) expressed on diverse innate immune cells, including NK cells, monocytes, macrophages, dendritic cells (DCs), and the C1q protein of the complement system [8,112,113] (Fig. 4a). FcγRs consist of three subtypes, which are FcγRI, FcγRII, and FcγRIII, among which the low-affinity FcγRs encompass activating receptors including FcγRIIa (CD32A), FcγRIIc (CD32C), FcγRIIIa (CD16A), FcγRIIIb (CD16B), alongside the inhibitory receptor FcγRIIb (CD32B). FcγRI (CD64) is the only high-affinity FcγR [114]. The equilibrium between the phosphorylation of the immunoreceptor tyrosine-based activation motif (ITAM) and FcγR immunoreceptor tyrosine-based inhibition motif (ITIM) determines Fc-mediated effector functions [115,116]. The engagement of the Fc regions of tumor-specific antibodies with FcγRIIIa receptors of NK cells leads to the formation of immunological synapses, triggering the phosphorylation of FcγRIIIa receptor's ITAM and subsequent downstream signaling events, which ultimately induces the secretion of cytotoxic granules comprising perforin and granzymes and effectively induce tumor cell death [117]. This phenomenon, known as antibody-dependent cellular cytotoxicity (ADCC), represents a particularly well-studied mechanism that enables targeted destruction of tumor cells [117,118]. Another mechanism, antibody-dependent target cell phagocytosis (ADCP), relies on phagocytic cells such as monocytes, macrophages, or neutrophils. Tumor-associated macrophages (TAM), for instance, primarily employ the activating FcγRIIa and FcγRIIIa receptors expressed on their surface to phagocytose tumor cells [119]. Antibody-mediated antitumor effects can also be attributed to complement-dependent cytotoxicity (CDC) or complement-dependent cellular cytotoxicity (CDCC) via activation of serum complements [8]. Initial binding of the soluble C1q to the Fc domain of antibodies can facilitate the assembly of C1q hexamers and stimulate a proteolytic cascade that leads to the activation of complement, which then produces a series of anaphylotoxins that can opsonize cells and regulate activity of immune cells. Moreover, the assembly of the Membrane-Attack-Complex (MAC) can directly lead to cellular destruction [120].

Multiple in vitro and in vivo studies have provided compelling evidence of Fc-mediated effects of clinically employed monoclonal antibodies used in cancer therapy [121,122]. In hematological malignancies, taking anti-CD20 rituximab as an example, Fc-mediated immune responses mainly contribute to the clearance of malignant lymphocytes. The rapid reduction observed in patient serum levels of complement component C2 subsequent to the administration of rituximab has offered indirect substantiation regarding the involvement of CDC [123]. Interestingly, combination with a macrophage checkpoint inhibitor, an anti-CD47 blocking monoclonal antibody's acquired resistance of rituximab was significantly reversed in patients with aggressive and indolent lymphoma, indicating that Fc-mediated phagocytosis by macrophages is an important mechanism of rituximab, and enhanced ADCP can help mitigate the therapeutic resistance [124]. CD47 expressing on tumor cells translates an antiphagocytic ‘don't eat me’ signal that suppresses phagocytosis by triggering SIRPα signaling in macrophages [125], and inhibition of CD47 can significantly augment a unique hyperphagocytic macrophage population (Fig. 4b).

In the field of solid tumors, taking anti-HER2 trastuzumab as an example, in addition to the blockade of HER2-mediated signals, engagement of innate immune cells greatly contributes to the reduction of tumor burden. Similar to rituximab, the blockade of its antitumor activity was demonstrated to rely on macrophage recruitment within the tumor tissues in a macrophage-depleted breast cancer xenograft model [126]. tumor-associated CD47 expression inversely correlates with survival in HER2-positive breast cancer patients, and complete tumor regression has been achieved after treatment with trastuzumab combined with CD47 inhibition in human HER2-positive breast cancer xenografts [127]. The co-administration of trastuzumab and an anti-CD47 mAb further exhibited increased susceptibility to ADCP in vitro against HER2-positive breast cancer cells that were already tolerant to trastuzumab [128]. Ex vivo assays utilizing NK cells obtained from breast cancer patients after treatment with trastuzumab have demonstrated enhanced ADCC stimulation against HER2-positive breast cancer cells [129]. Moreover, while trastuzumab alone has a minimal potential to induce CDC, a synergistic effect has been shown when it was used in combination with pertuzumab in vitro due to required binding and activation of the complement component 1q (C1q) [130]. Multiple in vivo studies have also provide evidence for the contribution of various myeloid subsets, including monocytes [131,132] and neutrophils [133,134] in Fc-mediated antitumor immune effects.

Modulating the binding affinity between the Fc domain and distinct FcγRs can significantly improve the clinical outcomes of monoclonal antibodies. Fc mediated effects can be optimized through two approaches: Fc glyco-engineering and Fc protein-engineering [135] (Fig. 4b). For instance, obinutuzumab is an anti-CD20 mAb binding to a distinct epitope of rituximab. With reduced fucosylation of the Fc domain, obinutuzumab exhibited enhanced ADCC and improved tumor inhibition in NHL compared to rituximab, and has been approved in 2013 for the treatment of the same diseases as rituximab [136]. Similarly, improved antitumor responses have been demonstrated in HER2-positive patients using glyco-engineering trastuzumab [137,138]. A study explored the potential association between FcγR polymorphisms and the clinical outcomes of breast cancer patients administrated with trastuzumab and found that the FcγRIIIa 158-Val/Val-and FcγRIIa 131-His/His-genotypes were independently correlated with improved objective response rate (ORR) and progression-free survival (PFS). The observed genotypes displayed a notably elevated Fc binding affinity and demonstrated enhanced Fc-mediated cytotoxicity [139]. Margetuximab is another classical anti-HER2 mAb that presents a comparable epitope as trastuzumab but distinguish itself with five amino acid substitutions (L235V/F243L/R292P/Y300L/P396L) in its Fc domain. The variant exhibits heightened affinity towards activating FcγRIIIa and diminished affinity towards inhibitory FcγRIIb [140]. Margetuximab has been shown to enhance ADCC in vitro, and induce enhanced HER2-specific immune responses in a xenograft model of HER2-amplified breast cancer using immune-deficient mice transgenic for human FcγRIIIa-158F [140,141]. Furthermore, margetuximab-treated patients have demonstrated higher circulating HER2-specific antibody levels and T cell responses compared to trastuzumab-treated patients [142]. The above engineering approaches also lead to enhanced ADCP. Designed with increased FcγRIIIa binding affinity, glyco-engineered trastuzumab has demonstrated heighted activity in monocytes and macrophages [143]. Similarly, engineered Fc variants of trastuzumab, possessing up to 70-fold greater FcγRIIa affinity, have shown enhanced phagocytosis by macrophages [144]. Novel strategies have also emerged to enhance the lysis of HER2-overexpressing tumor cells by γδT cells and NK cells, triggered by trastuzumab. A tribody construct, comprising two HER2-specific scFv domains fused to a fragment antigen binding domain directed toward FcγRIII expressed on γδT cells and NK cells, exhibited superior cytotoxic antitumor activity compared to trastuzumab in vitro [145].

### 4.2 T cell receptor mimic antibodies: recognition and selection of intracellular peptide/MHC complexes

T cell receptors (TCRs) consist of an α- and a β-chain that form a noncovalent association with the CD3 complex. After recognition and interaction of short peptides presented on the surface of tumor cells by major histocompatibility complex class I (MHC-I), the intracellular domain induces activation of T cells and subsequent release of cytotoxic molecules [146]. T cell receptor mimic (TCRm) antibodies can recognize the peptide/MHC complexes (pMHC) in a similar manner to TCRs but typically exhibit significantly stronger binding affinities compared to their TCR counterparts [147]. As they have an IgG antibody skeleton, the mechanisms of TCRm antibodies are similar to those of conventional monoclonal antibodies, including Fc-mediated immune effects, lysis of tumor cells through conjugation with toxins or drugs, and direct induction of tumor cell death [147,148].

Notably, as multiple intracellular targets such as transcription factors and neoantigens are not well accessible to cancer therapies, TCRm antibodies expand the range of therapeutic targets. A variety of cancer-specific short peptides have been identified and used to construct TCRm antibodies in experimental studies [149]. However, only a limited number of TCRm antibodies have entered clinical investigations, partly due to the difficulty of their generation via conventional techniques. Moreover, the exploration of novel intracellular targets supports their potential for constructions of other antibody-based agents, and the antigen recognition region of TCRm antibodies can be fused with the anti-CD3 scFv for construction of a bispecific T cell engager (BiTE) [150]. TCRm antibodies can also be engineered in the format of CARs. For instance, a TCRm antibody with exquisite selectivity and specificity for the AFP_158–166_ peptide complexed with human leukocyte antigen (HLA)-A*02:01 was selected and further used to develop AFP-specific CAR-T cells for the treatment of liver cancer [151]. The AFP CAR-T cells have also been involved in a clinical study (NCT03998033).

### 4.3 Bispecific T cell engagers: engagement of T-cell immune responses

A bispecific T cell engager (BiTE) consist of a single chain variable fragment specific to CD3 on T cell surface and a single chain variable fragment specific to a tumor antigen, which serves as a bridge between T cells and tumor cells independently from MHC restriction and costimulatory signals. After the formation of the immunologic synapse, activated T cells secret perforins and granzymes, which results in the cytotoxicity lysis of nearby tumor cells [152] (Fig. 4c). BiTEs have been extensively explored in multiple clinical trials, most of which utilize CD3 as one of the targets. Blinatumomab stands out as the first CD19 and CD3 bispecific BiTE available in the market, exhibiting remarkable clinical efficacy in the treatment of R/R child and adult B-ALL, as well as in patients with minimal residual disease (MRD)-positive B-ALL [153,154]. In addition to CD19, other tumor-associated antigens (TAAs) such as CD33, B-cell maturation antigen (BCMA), CD20, and CD123 have also shown potential for BiTEs in hematological malignancies [154]. There are also a variety of BiTEs undergoing clinical investigations that target TAAs overexpressed on solid tumors. For instance, pasotuxizumab is a BiTE targeting both prostate-specific membrane antigen (PSMA) and CD3 for the treatment of metastatic castration-resistant prostate cancer (mCRPC). Data of its phase I clinical trial have provide initial support for the application of BiTE antibodies in solid tumors [155,156]. BiTE antibodies against the class III variant of the epidermal growth factor receptor (EGFRvIII) for the treatment of glioblastoma (GBM) and delta-like ligand 3 (DLL3) for the treatment of SCLC have also shown promising outcomes in their phase I clinical investigations [154]. In multiple syngeneic solid tumor models, experiments have revealed that CD8+ T cells serve as both targets and mediators of BiTE activity, while CD4+ T cells have been identified to have an antagonistic role in BiTE efficacy. The local expansion of tumor-associated CD8+ T cells has been identified as a key factor for efficient lysis of tumor cells [157].

One crucial aspect in the development of BiTE antibodies is to extend their duration of action as their structural composition and molecular size lead to a short circulating half-life and repeated infusions are required during treatment. Engineered BiTEs fused to a silence Fc domain have shown prolonged half-life via interaction of FcRn, enabling enhanced infiltration of T cells into solid tumors. Such constructs are being explored as next-generation BiTEs in ongoing clinical trials. Glofitamab is a CD20 × CD3 T cell-engaging bispecific monoclonal antibody that have been approved for the treatment of adult patients with relapsed or refractory diffuse large B-cell lymphoma (DLBCL), in which the patients are ineligible to receive or cannot receive CAR T-cell therapy or have previously received CAR T-cell therapy [158,159]. Mosunetuzumab, which recently achieved approval from the European Medicines Agency (EMA), is also a full-length IgG BiTE against CD20-positive follicular lymphoma and has been well demonstrated in several clinical trials [160,161]. In addition to fused with monoclonal antibody fragment, an alternative approach designed multivalent T-cell engaging antibodies, which are engineered with three binding domains, anti-PSMA for tumor cell engagement, anti-albumin for half-life extension and anti-CD3 for T cell engagement. Initial clinical outcomes have shown their antitumor activity [162]. Moreover, in addition to the recruitment of T cells, novel bispecific and trispecific NK cell engagers have also been designed to activate NK cells and clear tumor cells via ADCC. The engagement between NK cells and target cells is primarily mediated by CD16, which is commonly expressed on NK cell surface [7,163].

Notably, therapeutic toxicity and resistance are both of considerable concern for clinical application of BiTEs. Cytokine release syndrome (CRS) and immune effector cell-associated neurotoxicity syndrome (ICANS) are commonly reported adverse events during the initial phases after BiTE infusion. CRS occurs following the activation of T cells and released inflammatory cytokines such as IL-6, while ICANS is associated with the adhension of T cells to cerebral microvascular endothelium, subsequent elimination of resident B cells, and ultimate excessive immune responses [154]. Strategies to address CRS mainly include symptomatic remission and anti-IL-6 therapy [164,165], while reducing the contact between T cells and epithelial cells is a crucial direction to prevent ICANS [154].

### 4.4 CAR-engineered immune cells: specific targeting and cellular toxicity towards tumor cells

Chimeric antigen receptors (CARs) are synthetic receptors that redirect T cells to recognize and attack tumor cells in a MHC-independent manner. CAR consists of an extracellular scFv recognition domain, a CD3ζ transmembrane domain and one or two stimulatory domains, commonly CD28 or 4-1BB, fused with an intracellular signaling domain from CD3ζ [166]. The interaction of the specific antigen by CARs initiates the formation of an immune synapse, which subsequently activates T cells to secrete perforin and granzyme and directly kill tumor cells [167,168] or induce apoptosis of target tumor cells via the TNF/TNFR or Fas/FasL pathways [169]. The secretion of inflammatory cytokines also contributes to the reshaping of the immunosuppressive TME [169,170]. CAR-T cell therapy has gained remarkable success in hematological malignancies, with six FDA-approved CAR-T cell products targeting either CD19 or BCMA [171]. Despite facing numerous challenges, CAR-T cells against solid tumors are also undergoing investigation in multiple clinical trials [172].

The intracellular signaling domains of CARs have received significant attention due to their influence on T cell activation and persistence [173]. The second and third generation CARs are currently the most employed, which incorporate one or two costimulatory domains, exhibiting enhanced antitumor cytotoxicity and improved CAR-T cell persistence compared to first-generation CARs with a single CD3ζ domain [174,175]. Different costimulatory domains can affect CAR-T cell properties such as metabolic pathways and memory pheotypes [167]. For instance, CD28 domain is associated with stronger cellular effectors but fewer memory properties [176], [177], [178]. Novel costimulatory domains such as the inducible T-cell costimulator (ICOS) has yielded enhanced in vivo antitumor effects and prolonged CAR-T cell persistence in conjunction with either CD28 or 4-1BB [179]. Engineering modifications of the CD3ζ signaling domain, via deletion or mutation of the ITAMs, have been shown to increase the activation threshold of CARs and modulate their affinity [178,180]. The hinge region of CARs is typically derived from the CH2-CH3 domain of IgG antibodies or the spacer domain of CD4 or CD8 [181]. The length of the hinge region is determined by the dimensions of the target tumor antigen, which affects its binding with the scFv domain [182]. Transmembrane regions derived from CD3ζ domains are commonly employed in CAR construction to ensure robust signaling due to their inherent activity [183]. Alterative transmembrane domains derived from CD28 have also been demonstrated to contribute to higher CAR expression and clonal expansion of CAR-T cells [184,185].

The affinity and specificity of the extracellular scFv domain directly determine the efficacy and therapeutic safety of CAR-T cells. An ideal CAR construction requires both high specificity and appropriate affinity to ensure recognition of tumor cells with low expression level of targeted antigens without “on-target, off-tumor” toxicity [186], [187], [188]. Engineering strategies to improve antitumor efficacy and safety of CAR-T cells include affinity tuning of CARs and logic-gated CAR expression and activation. Affinity tuning and modifying of the scFv domain can be accomplished by construction of a diversity of mutations and subsequent affinity screening [189,190]. Our team have established libraries of immune cells that display diverse repertoires of CARs. Based on scFv libraries with high diversity, the synthetic libraries consist of 10^6^ murine or human CAR clones, which can be displayed on genetically modified immune cells for both adoptive cellular therapy and discovery of novel targets for cancer immunotherapies [191]. Similar to bispecific antibodies, engineering T cells to express bispecific CARs or two different CARs is a typical OR-logic gated circuit that allows for T cell activation in the present of either target, which improve the efficacy of CAR-T cells. In order to improve CAR-T cell specificity, conditional CAR expression and activation controlled by another receptor or environmental factors aim to prevent damage of normal cells with low expression level of targeted antigens. NOT-logic CAR T cells, on the other hand, incorporate an inhibitory CAR to suppress CAR activation when encountering non-specific targets. These engineering strategies have been comprehensively reviewed in recent literature [186].

Interestingly, the combination of BiTEs with CAR-T cells has emerged as an intriguing approach to address the challenge of tumor heterogeneity. Through the engineering of EGFRvIII-specific CAR-T cells to express BiTEs against EGFR, a novel CAR-T cell platform known as CART.BiTE can redirect CAR-T cells and recruit untransduced bystander T cells to effectively target wild-type EGFR. Notably, CART.BiTE cells exhibited superior antitumor activity compared to single EGFRvIII-specific CAR-T cells in mouse models of glioblastoma without detectable toxicity [192]. Similarly, genetically engineered macrophages expressing EGFRvIII-specific BiTEs and IL-12 also led to a notable decrease in early tumor burden in both subcutaneous and intracranial mouse models of GBM [193]. Actually, the primary objective of combining BiTEs with CAR-T cells is to transform the immunosuppressive TME to create a conductive milieu for enhanced antitumor immune responses. Furthermore, the exploration of alternative immune cell subsets, such as NK cells, has gained considerable attention in CAR construction, and promising clinical-stage demonstrations of novel CAR-engineered immune cells provide a broad platform for the development of next-generation immune cell therapies [194].

### 4.5 Antibody-cytokine fusion proteins: activation of the proinflammatory TME

In contrast to targeted antibody and cellular immunotherapy, though cytokines is an important component of the immune system and exert powerful immunomodulatory effects, the uncontrolled immune effects including toxicity and lack of efficacy have long limited their application in cancer therapy [195]. However, combing cytokines with antibody engineering has the potential to greatly enhance their immunomodulatory role (Fig. 4e). A variety of antibody-cytokine fusion proteins have been developed based on full-length IgG and antibody fragments [196].

By fusing with a Fc fragment, antibody-cytokine proteins exhibit extended half-life compared to soluble cytokines. For example, interleukin-2 (IL-2), the earliest and most extensively studied cytokine, has been the subject of various antibody IL-2 fusion proteins, including fusion with intact IgG, scFv, or an Fc domain. These fusion proteins enable the production of higher local cytokine concentrations, reduction of effective doses, and prolongation of IL-2 half-life [20]. One prominent example is the fusion of ganglioside 2 (GD2)-specific IgG antibody with IL-2 at the C-terminal of the heavy chain. This novel construct has successfully advanced into phase II clinical trials for the therapeutic management of neuroblastoma [197]. Furthermore, an IL-2 variant with a D20T mutation fused to an antibody targeting the necrotic core of tumors (NHS-IL2LT) has been demonstrated in immune-competent mice and cynomolgus monkeys [198]. The IgG-IL-2 fusion protein exhibits improved pharmacokinetic properties, as evidenced by an increased half-life from 7.8 to 11 h, comparable to an IgG antibody [199]. Significantly, the antibody-IL-2 fusion protein NHS-IL2LT exhibited remarkable efficacy in a mouse model of Lewis lung carcinoma (LLC), leading to its assessment as a monotherapy in patients with advanced solid tumors through a phase I/II clinical study [200]. Another IL-2 mutant, namely FSD13, was found to possess superior potency compared to the wild-type human IL-2. FSD13 demonstrated proficient activation of CD4+ T cells, CD8+ T cells, and NK cells, accompanied by reduced adverse side effects and efficacy in inhibiting tumor growth [201]. Additional cytokines, such as IL-12, granulocyte-macrophage colony-stimulating factor (GM-CSF), and interferon-γ (IFN-γ), have also been redeveloped as fusion proteins. These antibody-cytokine fusion constructs, commonly referred to as “immunocytokines”, have gained recent attention in numerous clinical trials [195]. Immunocytokines surpass the ability in stimulating the antitumor immune cell functions, simultaneously exering reduced systemic toxicity compared to wild-type cytokines. As a result, they are described as the next generation of cytokine products [195].

## 5 Discussion

We have summarized antibody-based drugs that have been approved by the FDA for cancer therapy over the years in Table 1. In terms of molecular structure, most of them are humanized monoclonal antibodies, of which IgG1 and IgG4 are commonly used subtypes. In recent years, ADCs have developed rapidly, accounting for a considerable proportion. In terms of targets, lineage specific epitopes such as CD20 are generally selected for hematological malignancies, while overexpressed tumor-associated antigens (TAA) such as HER2 are commonly used for solid tumors. The discovery of immune checkpoints provided a new direction for the selection of targets for antibodies against solid tumors. However, although antibody therapy is an important aspect of cancer therapy and have been steadily developing, limited therapeutic responses, drug resistance, as well as therapeutic side effects are all issues that need to be considered.

**Table 1:** **Antibody drugs approved by the FDA over the years**.

Antibody name	Target	Mechanism of action (drugbank)	Antibody type	Cancer type	Approved year
Rituximab	CD20	1) direct signaling, complement-mediated cytotoxicity (CDC)	IgG1 κ chimeric murine/human monoclonal antibody	chronic lymphocytic leukemia follicular lymphoma non-Hodgkin's lymphoma	1997
		2) antibody-dependent cell-mediated cytotoxicity (ADCC)			
Trastuzumab	epidermal growth factor receptor 2 (HER2)	1) binds to the extracellular ligand-binding domain and blocks the cleavage of the extracellular domain of HER2 and inhibits HER2-mediated intracellular signaling cascades	recombinant IgG1 κ, humanized monoclonal antibody	breast cancer gastric cancer	1998
		2) antibody-dependent cell-mediated cytotoxicity (ADCC)			
Gemtuzumab ozogamicin	CD33	after internalization of antibody-CD33 complex, the calicheamicin derivative is released inside the lysosomes of the myeloid cell, then the released calicheamicin derivative binds to DNA in the minor groove resulting in site-specific DNA double strand breaks via formation of a p-benzene diradical and eventually cell death is induced	recombinant humanized IgG4 κ antibody conjugated with calicheamicin derivative	acute myelogenous leukemia	2000
Alemtuzumab	CD52	1) antibody-dependent cellular cytolysis	recombinant IgG1 κ, DNA-derived humanized monoclonal antibody	chronic lymphocytic leukemia	2001
		2) complement-mediated lysis			
		3) repopulation of lymphocytes			
Ibritumomab tiuxetan	CD20	after recognition of CD20 epitope on B cells, the radioactive yttrium to destroy the cell via production of beta particles	Indium or yttrium conjugated murine IgG1 kappa monoclonal antibody	non-Hodgkin's lymphoma	2002
Cetuximab	epidermal growth factor receptor (EGFR)	1) competitively inhibits the binding of epidermal growth factor (EGF) and other ligands to inhibit the EGFR signaling pathway	IgG1 chimeric murine/human monoclonal antibody	colorectal cancer head and neck cancer	2004
		2) inhibits cell growth and induces cell apoptosis			
		3) decreases matrix metalloproteinase and vascular endothelial growth factor (VEGF) production			
		4) internalization of the antibody-receptor complex and an overall downregulation of EGFR expression			
Bevacizumab	vascular endothelial growth factor A (VEGF-A)	used in combination with antineoplastic agents, competitively inhibits the binding of circulating vascular endothelial-derived growth factor (VEGF) to their receptors, then prevents formation of new blood vessels, decreases tumor vasculature, and reduces tumor blood supply	humanized monoclonal IgG antibody	breast cancer cervical cancer colorectal cancer glioblastoma liver cancer non-small cell lung cancer ovarian cancer glioma renal cell carcinoma	2004
Panitumumab	epidermal growth factor receptor (EGFR)	1) competitively inhibits the binding of epidermal growth factor (EGF) and other ligands to inhibit the EGFR signaling pathway	recombinant human IgG2 monoclonal antibody	colorectal cancer	2006
		2) inhibits cell growth and induces cell apoptosis			
		3) decreases pro-inflammatory cytokine and vascular endothelial growth factor (VEGF) production			
		4) internalization of the antibody-receptor complex and an overall downregulation of EGFR expression			
Ofatumumab	CD20	1) binds to the small and large extracellular loops of CD20	IgG1 κ human monoclonal antibody	chronic lymphocytic leukemia	2009
		2) complement-dependent cytotoxicity (CDC)			
		3) antibody-dependent cellular cytotoxicity (ADCC)			
Denosumab	receptor activator of nuclear factor kappaB ligand (RANKL)	prevents RANKL from activating its receptor RANK, inhibits osteoclast formation, function, and survival, thereby decreasing bone resorption and increasing bone mass and strength in both cortical and trabecular bone	human IgG2 monoclonal antibody	bone cancer	2010
Brentuximab vedotin	CD30	after internalization of antibody-CD30 complex, the monomethyl auristatin E (MMAE) is released, binding of MMAE to tubulin disrupts the microtubule network within the cell, inducing cell cycle arrest and cell apoptotis	chimeric human-murine IgG1 conjugated with monomethyl auristatin E (MMAE)	Hodgkin's lymphoma T-cell lymphoma peripheral T-cell lymphoma anaplastic large cell lymphoma primary cutaneous anaplastic large cell lymphoma	2011
Aflibercept	vascular endothelial growth factor (VEGF)	competitively prevents vascular endothelial growth factor-A (VEGF-A) and placental growth factor (PIGF) to binding to receptors, suppresses neovascularization and decrease vascular permeability	recombinant protein composed of the binding domains of human vascular endothelial growth factor receptor 1 (VEGFR1) and VEGFR2, fused with the Fc region of human IgG1	colorectal cancer	2011
Ipilimumab	cytotoxic T-lymphocyte antigen 4 (CTLA4)	prevents the inhibition of T-cell mediated immune responses to tumors	humanized IgG1 monoclonal antibody	liver cancer non-small cell lung cancer mesothelioma malignant melanoma renal cell carcinoma	2011
Pertuzumab	human epidermal growth factor receptor 2 (HER2)	1) binds to the extracellular ligand-binding domain and blocks the cleavage of the extracellular domain of HER2 and inhibits HER2-mediated intracellular signaling cascades	recombinant humanized monoclonal antibody	breast cancer	2012
		2) antibody-dependent cell-mediated cytotoxicity (ADCC)			
Mogamulizumab	C-C chemokine receptor type 4 (CCR4)	1) block CCR4-mediated cellular migration, proliferation of T cells, and angiogenesis	humanized monoclonal antibody	adult T-cell leukemia/lymphoma cutaneous T-cell lymphoma peripheral T-cell lymphoma	2018
		2) depletes Treg cells			
		3) antibody-dependent cell-mediated cytotoxicity (ADCC)			
Trastuzumab emtansine	human epidermal growth factor receptor 2 (HER2)	1) after internalization of antibody-HER2 complex, the DM1 is released, binding of DM1 to tubulin disrupts the microtubule network within the cell, inducing cell cycle arrest and cell apoptotis	humanized IgG1 conjugated with DM1	breast cancer	2013
		2) antibody-dependent cell-mediated cytotoxicity (ADCC)			
Obinutuzumab	CD20	1) direct cytotoxic effect	humanized monoclonal antibody	chronic lymphocytic leukemia follicular lymphoma non-Hodgkin's lymphoma	2013
		2) antibody-dependent cell-mediated cytotoxicity (ADCC) higher than classic rituximab			
Blinatumomab	CD19, CD3	bridges T-cells and tumor cells together, induces an immune response that leads to T-cell activation and proliferation	bispecific T-cell engager formed by the recombinant fusion of an anti-CD3 single-chain variable fragment (scFv) and an anti-CD19 scFv	B-cell acute lymphoblastic leukemia	2014
Pembrolizumab	programmed cell death 1 (PD-1)	antagonizes PD-1 interaction with its known ligands PD-L1 and PD-L2, prevents the inhibition of TCR-mediated T-cell proliferation and cytokine production	IgG4 kappa humanized monoclonal antibody	breast cancer cervical cancer colorectal cancer esophageal cancer gastric cancer head and neck cancer liver cancer non-small cell lung cancer diffuse large B-cell lymphoma Hodgkin's lymphoma pancreatic cancer squamous cell carcinoma urogenital cancer malignant melanoma	2014
Ramucirumab	vascular endothelial growth factor receptor 2 (VEGFR-2)	prevents binding of VFGFR2 to its ligands, prevents VEGF-stimulated receptor phosphorylation and downstream ligand-induced proliferation, permeability, and migration of human endothelial cells	IgG1 humanized monoclonal antibody	colorectal cancer gastric cancer liver cancer non-small cell lung cancer	2014
Nivolumab	programmed cell death 1 (PD-1)	antagonizes PD-1 interaction with its known ligands PD-L1 and PD-L2, prevents the inhibition of TCR-mediated T-cell proliferation and cytokine production	IgG4 humanized monoclonal antibody	colorectal cancer esophageal cancer gastric cancer head and neck cancer non-small cell lung cancer Hodgkin's lymphoma mesothelioma squamous cell carcinoma urogenital cancer malignant melanoma renal cell carcinoma	2014
Necitumumab	epidermal growth factor receptor (EGFR)	1) competitively inhibits the binding of epidermal growth factor (EGF) and other ligands to inhibit the EGFR signaling pathway	IgG1 recombinant humanized monoclonal antibody	non-small cell lung cancer	2015
		2) internalization of the antibody-receptor complex and an overall downregulation of EGFR expression			
Atezolizumab	programmed cell death 1 (PD-1)	prevents the interaction of PD-L1 and PD-1, removes inhibition of immune responses	humanized monoclonal antibody	breast cancer liver cancer non-small cell lung cancer small cell lung cancer urogenital cancer malignant melanoma	2016
Inotuzumab ozogamicin	CD22	after drug-CD22 complex is internalized into the cell, released calicheamicin derivative mediates apoptosis of the cell by binding to the minor groove of DNA	IgG4 humanized monoclonal conjugated with calicheamicin derivative	B-cell acute lymphoblastic leukemia	2017
Durvalumab	programmed cell death ligand 1 (PD-L1)	binds to PD-L1 and prevents its association with PD-1 and CD80, activates the immune responses mediated by cytotoxic T cells	IgG1 κ humanized monoclonal antibody	non-small cell lung cancer small cell lung cancer	2017
Avelumab	programmed cell death ligand 1 (PD-L1)	binds to PD-L1 and prevents its association with PD-1 and CD80, activates the immune responses mediated by cytotoxic T cells	IgG1 λ humanized monoclonal antibody	merkel cell carcinoma urogenital cancer renal cell carcinoma	2017
Moxetumomab pasudotox	CD22	after binding to CD22 and internalization, released toxin inhibits protein translation which induces an apoptotic state of the high CD22-expressed cancer cell	recombinant immunotoxin in which a stabilized Fv segment is fused to the Pseudomonas exotoxin A (PE38)	hairy cell leukemia	2018
Cemiplimab	programmed cell death 1 (PD-1)	prevents the interaction of PD-L1 and PD-1, inhibits PD-1 mediated suppression of T cell activity	humanized monoclonal antibody	basal cell carcinoma non-small cell lung cancer squamous cell carcinoma	2018
Trastuzumab deruxtecan	human epidermal growth factor receptor 2 (HER2)	after binding to HER2 and internalization, released deruxtecan crosses cell membranes, causes targeted DNA damage and cell apoptosis	IgG1 humanized monoclonal antibody conjugated with deruxtecan	breast cancer gastric cancer	2019
Enfortumab vedotin	nectin cell adhesion molecule 4 (Nectin 4)	after binding to Nectin 4 and internalization, released MMAE disrupts the microtubule network within the cell, arresting the cell cycle and ultimately inducing apoptosis	humanized monoclonal antibody conjugated with monomethyl auristatin E (MMAE)	urogenital cancer	2019
Polatuzumab vedotin	CD79b	after binding to CD79b and internalization, released MMAE disrupts the microtubule network within the cell, arresting the cell cycle and ultimately inducing apoptosis	humanized monoclonal antibody conjugated with monomethyl auristatin E (MMAE)	diffuse large B-cell lymphoma	2019
Sacituzumab govitecan	trophoblast cell-surface antigen 2 (TROP-2)	binding of anti-TROP-2 monoclonal antibody to TROP-2 results in rapid internalization, released SN-38 inhibits DNA topoisomerase I, leading to DNA damage and eventual cell death	humanized monoclonal antibody conjugated with topoisomerase I inhibitor SN-38	breast cancer urogenital cancer	2020
Margetuximab	human epidermal growth factor receptor 2 (HER2)	similar to trastuzumab, with a modified Fc region encoding five amino acid substitutions (L235V, F243L, R292P, Y300L, and P396L) to alter Fc receptor binding, results in increased ADCC	Fc-engineered human/mouse chimeric IgG1κ monoclonal antibody	breast cancer	2020
Tafasitamab	CD19	an IgG1/2 hybrid Fc-domain has been modified with 2 amino acid substitutions to enhance its cytotoxicity	IgG1/2 recombinant humanized monoclonal antibody	diffuse large B-cell lymphoma	2020
		1) direct apoptosis			
		2) immune-mediated effector			
Naxitamab	GD2 ganglioside (GD2)	co-administration with GM-CSF	IgG1 humanized monoclonal antibody	neuroblastoma	2020
		1) immune-mediated effector: ADCC, CDC			
Dostarlimab	programmed cell death 1 (PD-1)	binds to PD-1 and prevents interactions with PD-L1 and PD-L2, allowing the anti-tumor immune response to proceed unimpeded	IgG4 humanized monoclonal antibody	endometrial cancer solid cancer	2021
Amivantamab	epidermal growth factor receptor (EGFR), mesenchymal-epithelial transition factor (MET)	1) Fc portion contains 90% less fucose than normal antibodies for increased binding to the FcγRIIIa region, enhanced immune-mediated effector	humanized bispecific antibody	non-small cell lung cancer	2021
		2) prevents ligands from binding to EGFR and MET and blocks signaling			
		3) EGFR and MET on the cell surface are internalized and downregulates the expression of EGFR and MET on NSCLC cell surfaces			
Loncastuximab tesirine	CD19	following binding to CD19 and internalization into the cell, released SG3199, a pyrrolobenzodiazepine (PBD) dimer cytotoxin, binds to the DNA minor groove, forming cytotoxic DNA interstrand crosslinks, leading to B-cell cell death	IgG1 humanized monoclonal antibody conjugated with a pyrrolobenzodiazepine (PBD) dimer cytotoxin	diffuse large B-cell lymphoma	2021
Tisotumab vedotin	tissue factor (TF)	1) after binding and internalization, released MMAE disrupts the microtubule network of actively dividing cells, leading to cell cycle arrest and apoptotic cell death	IgG1 humanized monoclonal antibody conjugated with monomethyl auristatin E (MMAE)	cervical cancer	2021
		2) immunogenic cell death			
		3) Fcγ receptor-mediated effector functions			
Relatlimab	lymphocyte activation gene 3 (LAG-3)	in combination with Nivolumab	IgG4 humanized monoclonal antibody	melanoma	2022
		1) LAG-3 expression is tied to continuous antigen exposure and T cell exhaustion, antagonism of LAG-3 promotes T-cell proliferation and cytokine secretion, restores tumor immunosurveillance			
		2) potentiate the anti-tumor effects of PD-1 blockade			
Tebentafusp	CD3, glycoprotein 100 (GP100)	the TCR arm binds to a gp100 peptide bound to HLA-A, the anti-CD3 effector domain engages and activates T cells	bispecific T-cell engager consists of a TCR targeting domain or a TCR arm fused to an anti-CD3 scFv	uveal melanoma	2022
Mosunetuzumab	CD20, CD3	simultaneously bound to CD20 on B-cells and CD3 on T-cells, recruits T-cells and leads to their activation, which ultimately induces B-cell lysis and cell death	humanized bispecific antibody	non-Hodgkin's lymphoma	2022
Tremelimumab	cytotoxic T lymphocyte-associated antigen-4 (CTLA-4)	blocks the interaction of CTLA-4 with CD80 and CD86, limiting its negative regulatory effect on T cell activation	IgG2 humanized monoclonal antibody	unresectable hepatocellular carcinoma (uHCC)	2022
Mirvetuximab soravtansine	folate receptor α (FRα)	1) after binding to FRα and internalization, released DM4 disrupts the microtubule network within the cell, leading to cell cycle arrest and apoptosis	IgG1 humanized monoclonal antibody conjugated with genotoxic compound DM4	ovarian cancer	2022
		2) diffuse across cell membranes and lead to the death of neighboring antigen-negative cells			
Epcoritamab	CD3, CD20	by targeting both CD3 and CD20, promotes the release of proinflammatory cytokines and the lysis of CD20+ malignant B-cells through selective T-cell–mediated cytotoxic activity	IgG1 humanized bispecific T-cell engager	B-cell non-Hodgkin lymphoma	2022
Glofitamab	CD3, CD20	binds bivalently to CD20 and monovalently to CD3, creating an immunological synapse that serves to recruit T-cells to CD20-expressing B-cells, allows for potent T-cell activation and proliferation which ultimately results in the lysis of the target B-cells	bispecific T-cell engager	B-cell non-Hodgkin lymphoma	2023

Clinical resistance is an important cause of partial reaction and cancer relapse. Innate resistance is determined by the intra-tumor heterogeneity (ITH), which stems from genomic alterations via diverse mutational mechanisms. The selective therapeutic pressure imposed by antibodies drives the acquired resistance, which is shown as eradication of targeted cellular clones, the acquisition of novel mutations conferring resistance, and adaptive responses in signaling pathways and epigenetic mechanisms, as well as elevated production of inhibitory molecules that impair immune cell functions [202]. Impairments in antibody-receptor interactions and alterations in receptor structure also attribute to monoclonal antibody-mediated resistance [203]. For example, the most frequently reported mechanisms associated with cetuximab and trastuzumab resistance involve genetic mutations in downstream signaling pathways, along with the overexpression of other surface receptors [204,205]. Epithelial to mesenchymal transition (EMT) represents a cellular phenomenon in which epithelial cells undergo a phenotypic and behavioral transition toward a mesenchymal state, accompanied by the downregulation of epithelial characteristics. This transformative process empowers cells with enhanced migratory capacity and invasion properties [206]. EMT has been demonstrated to contribute to the resistance of several classical antibodies, including cetuximab and trastuzumab [207,208]. Another resistance mechanism involves the impaired immune effectors mediated by NK cells and T cells. Similar to T cells, NK cells also express immune checkpoints, such as T cell immunoglobulin and ITIM domain (TIGIT), and blockade of TIGIT has been shown to augment responses to trastuzumab [209]. As for T cells, tumor evolution and deficient immune environment can lead to weakened effects of ICIs and persistent immune cell damage [102].

There are multiple approaches to address targeted resistance: 1) Improve the immune effects via molecular engineering and structural modulation. Through glyco-engineering of the CH2 domain and amino acid mutations of the Fc region, Fc-mediated immune effectors and engagement can be enhanced or reduced. There have been multiple studies that have demonstrated certain mutations associated with enhanced ADCC, ADCP, and CDC, as well as increased half-life and coengagement [135]. Besides IgG subtype, IgA and IgM have gained attention for antibody therapy. IgA antibodies have demonstrated superior binding to its FcRs and synergistic efficacy with IgG [210,211] and IgM antibodies also exhibit strong antigen-binding avidity, rendering them highly effective in binding repetitive epitopes and antigens with low expressing levels. A multivalent agonistic IgM antibody with ten binding sites to DR5 demonstrated greater potency than a corresponding agonistic DR5 IgG antibody [212]. 2) Improve antibody selectivity to simultaneously target distinct targets. Bispecific and multispecific antibodies have been designed to simultaneously target two or more antigens that independently or synergistically drive tumor growth and such bispecific antibodies have also been approved for clinical treatment. Moreover, potential novel targets and non-druggable targets provide rich resources for antibody construction, with the help of chemical tools and genetic approaches [213]. With the development of proteomics and chemoproteomics approaches, innovative strategies are emerging for target screening, identify and validation [214,215], and different antibodies targeting alternative protein conformations, binding sites, and resistance-related pathways may help address the variety of resistance mechanisms [202]. 3) Improve tissue permeability. None of the approved antibodies has achieved sufficient delivery across the blood-brain barrier, with levels in the brain corresponding to only 0.01%–0.1% of those in plasma. It is a key challenge in the development of monoclonal antibodies to treat central nervous system and prevent intracerebral metastases [216]. Invasive and non-invasive technologies have been well reviewed elsewhere [217]. Notably, biological nanoparticles, the small extracellular vesicles (sEVs), for example, exhibit promising potential to assist antibody therapy as they have superior biological properties, such as appropriate size and good bioavailability, which can lead to enhanced infiltration of the payloads and mitigation of adverse reactions [218]. 4) Combination therapy. Actually, a more practical trend and widely used option is to employ antibodies as combinations with chemotherapy, radiation, tyrosine kinase inhibitors, vaccines, cellular therapies, as well as antibodies targeting other antigens or epitopes. For instance, the immunosuppressive TME poses challenges for ICIs mainly due to inadequate inflammatory responses [219]. The combination of antibody therapy with cellular immunotherapy has exhibited enhanced efficacy. The administration of adaptive CAR-T cells may lead to more extensive infiltration of activating T cells, while simultaneous blockade of PD-1/PD-L1 signaling can contribute to the issue of T cell exhaustion, which is one of the obstacles of CAR-T cell therapy. CAR-T cells engineered to express full-length anti-PD-1 antibodies have shown partial response and promising survival outcome in a patient with refractory epithelial ovarian cancer (EOC) [220]. 5) Development of therapeutic antibody fragments and novel agents. Based on a detailed understanding of antibody structure, various domains such as scFv and nanobodies and conjugation agents like ADCs and fusion proteins fully extend the structural potency of monoclonal antibodies [221]. Nanobodies are antibody fragments derived from heavy-chain only IgG antibodies, with promising properties including smaller size, high antigen affinity, and remarkable stability in extreme conditions, thus having the potential to address some of the obstacles of conventional monoclonal antibodies. With the approval of the first nanobody, Caplacizumab, for the treatment of other diseases, many nanobodies for cancer therapy are still undergoing their clinical investigations [222].

## 6 Perspective

Just as Paul Ehrlich envisioned a century ago [223], the broad potential of immunoglobulins in cancer therapy have made them the “promised land”, which “will yield rich treasures for biology and therapeutics”. While IgG monoclonal antibodies remain the predominant avenue in antibody drug development and approval, recent advancements in technology platforms have paved the way for substantial progress in bispecific antibodies and ADCs, as well as novel antibody fragments including nanobodies and antibody fusion proteins. It is worth noting that the current landscape of target selection in global research exhibits a relative limitation. The development of new drug target discovery strategies, such as artificial intelligence (AI) and the therapeutic transformation of non-druggable targets, holds great promise in expanding the repertoire of potential antibody drug targets. Additionally, the synergistic combination of chemotherapeutic drugs and cellular therapies presents a clinically viable approach for enhancing the efficacy of antibody monotherapy.

## Declaration of competing interest

The authors declare that they have no conflicts of interest in this work.
